# Viscoelastic Properties of Hyaluronan in Physiological Conditions

**DOI:** 10.12688/f1000research.6885.1

**Published:** 2015-08-25

**Authors:** Mary K. Cowman, Tannin A. Schmidt, Preeti Raghavan, Antonio Stecco

**Affiliations:** 1 Biomatrix Research Center, Department of Chemical and Biomolecular Engineering, Polytechnic School of Engineering, New York University, New York, NY, 10010, USA; 2 Faculty of Kinesiology & Schulich School of Engineering - Centre for Bioengineering Research & Education, University of Calgary, Calgary, AB, T2N 1N4, Canada; 3 Department of Rehabilitation Medicine, Rusk Rehabilitation, New York University School of Medicine, New York, NY, 10016, USA; 4 Department of Internal Medicine, University of Padova, Padua, 35100, Italy

**Keywords:** hyaluronan, viscosity, viscoelasticity, lubrication, fascia

## Abstract

Hyaluronan (HA) is a high molecular weight glycosaminoglycan of the extracellular matrix (ECM), which is particularly abundant in soft connective tissues. Solutions of HA can be highly viscous with non-Newtonian flow properties. These properties affect the movement of HA-containing fluid layers within and underlying the deep fascia. Changes in the concentration, molecular weight, or even covalent modification of HA in inflammatory conditions, as well as changes in binding interactions with other macromolecules, can have dramatic effects on the sliding movement of fascia. The high molecular weight and the semi-flexible chain of HA are key factors leading to the high viscosity of dilute solutions, and real HA solutions show additional nonideality and greatly increased viscosity due to mutual macromolecular crowding. The shear rate dependence of the viscosity, and the viscoelasticity of HA solutions, depend on the relaxation time of the molecule, which in turn depends on the HA concentration and molecular weight. Temperature can also have an effect on these properties. High viscosity can additionally affect the lubricating function of HA solutions. Immobility can increase the concentration of HA, increase the viscosity, and reduce lubrication and gliding of the layers of connective tissue and muscle. Over time, these changes can alter both muscle structure and function. Inflammation can further increase the viscosity of HA-containing fluids if the HA is modified via covalent attachment of heavy chains derived from Inter-α-Inhibitor. Hyaluronidase hydrolyzes HA, thus reducing its molecular weight, lowering the viscosity of the extracellular matrix fluid and making outflow easier. It can also disrupt any aggregates or gel-like structures that result from HA being modified. Hyaluronidase is used medically primarily as a dispersion agent, but may also be useful in conditions where altered viscosity of the fascia is desired, such as in the treatment of muscle stiffness.

## Introduction

Hyaluronan (HA) is a high molecular weight glycosaminoglycan polymer of the extracellular matrix (ECM) in vertebrate tissues ^1^. It is composed of disaccharides of alternating D-glucuronic acid and N-acetyl D-glucosamine connected by β-1,3 and β-1,4 glycosidic bonds, respectively. In most healthy tissues, HA has an average molecular weight of approximately 6–8 million ^2^. HA has a high turnover rate, but homeostasis is normally maintained by similar rates of synthesis and degradation ^3, 4^. It can be enzymatically cleaved by hyaluronidases, or chemically degraded by hydroxyl radicals and peroxynitrite during inflammation ^5– 7^. It has a wide variety of physiological functions in the mammalian body, including maintenance of a viscoelastic cushion to protect tissues, control of tissue hydration and water transport, lubrication of biointerfaces, creation of large assemblies with proteins and proteoglycans in the ECM, and receptor-mediated signaling roles in cell detachment, mitosis, migration, tumor development, and inflammation ^3, 8, 9^. HA is ubiquitous, but is particularly abundant in soft connective tissues, including between deep fascia and muscle, within muscle ^10– 12^, and also between the collagen layers that compose the deep fascia. This tissue is a multilayered structure formed by two to three layers of densely packed collagen fibers ^13, 14^, spaced by a layer of loose connective tissue (containing adipose cells, sulfated glycosaminoglycans and HA) ^15– 17^. The proposed function of HA is to facilitate smooth gliding between these structures during movement, and in the transmission of force generated from muscle contraction.

The aim of the present study was to examine more closely the viscoelastic properties of HA in association with these structures, and to evaluate if the above mechanisms can be affected by HA viscoelastic variations.

## Review of the field

Solutions of high molecular weight hyaluronan can be highly viscous with non-Newtonian flow properties (see for example the review by Cowman and Matsuoka ^18^ and references therein). These properties may affect the movement of HA-containing fluid layers within and underlying the deep fascia. Additionally, the concentration and molecular weight of HA affects its contribution to the lubrication of biological interfaces. Changes in the concentration, molecular weight, or even covalent modification of HA in inflammatory conditions, as well as changes in binding interactions with other macromolecules, can have dramatic effects on the sliding movement of fascia.

### High molecular weight and the semi-flexible chain of HA are key factors leading to the high viscosity of dilute solutions

For a semi-flexible polymer such as HA, the volume occupied by each chain is very large. Most of the volume is water, not bound by the polymer, and the polymer shape is constantly changing, but the water still contributes to the effective size of each molecule because the solvent movement is affected by frictional interaction with closely spaced polymer segments. Due to its rapid chain motions, the time-average shape of the molecule can be described as a sphere, with greatest density of chain segments near the center. Furthermore, the effective sphere-like volume of a wormlike HA chain in a good solvent grows approximately as the molecular weight raised to the power of 1.8 (=M ^1.8^). This means that, the larger the polymer, the lower the average density because the volume grows faster than the mass. For HA, with molecular weight normally in the millions, this leads to extremely large chain volumes. In contrast, the volume of a compactly folded globular protein chain increases only in direct proportion to the number of amino acids and is therefore proportional to the molecular weight to the first power. The expanded shape of a flexible polymer in solution is a key reason for the high viscosity of "unfolded" polymer solutions.

The hydrodynamic volume of HA chains is usually studied at an ionic strength that is close to physiological. At that ionic strength, the charges due to the carboxylate groups on the HA chain are almost completely screened from each other, and the repulsion between them does not significantly expand the coil volume. In solutions with lower salt concentrations than about 0.15 M NaCl, the electrostatic repulsion would increase the hydrodynamic volume of individual HA molecules, and also increase repulsion between molecules.

The specific viscosity, *η _sp_*_,_ of an ideal polymer solution is proportional to the fraction of the solution volume that is filled with polymer chains. The Stokes-Einstein equation expresses the specific viscosity of a dilute solution of spherical particles (determined from the solution viscosity, *η*, and that of the pure solvent, *η _0_*) as proportional to the product of the number of spherical particles per unit of solution volume, *n*, and the volumes of the particles themselves, *V*. This product corresponds to the volume occupied by all the particles, divided by the solution volume, or the volume fraction, *φ*, of the solution that is occupied by particles. The occupied volume fraction can also be expressed in terms of the mass concentration of the polymer ( *c*, in g polymer/cm ^3^ of solution) multiplied by the specific volume of the polymer (in cm ^3^ occupied/g). The specific volume (inverse of the density) is proportional to the intrinsic viscosity *[η]*. As discussed above, the density of the polymer domain decreases with increasing molecular weight, so the intrinsic viscosity is a sensitive measure of the molecular weight. For HA in neutral aqueous salt solution at physiological ionic strength, the intrinsic viscosity is proportional to M ^0.8^ ^19^.

$$\eta_{sp}=\frac{\eta -\eta_{0}}{\eta_{0}}=\frac{\eta }{\eta_{0}}-1=2.5nV=2.5\varphi =c[\eta ]\;\text{in dilute solution}\;(1)$$

From Equation 1, we can see that the occupied volume fraction, *φ*, is equal to *0.4c[η]*. When the product *c[η]* is 2.5, the volume fraction is 1, and the solution can be considered to reach the "coil overlap" point. This is the nominal point at which the chains fill the solution and are forced to touch each other, although they already interact at lower concentrations/hydrodynamic volumes, and can interpenetrate at higher concentrations/hydrodynamic volumes because the coil volumes contain mostly solvent. An ideal solution should be much more dilute than the critical concentration for coil overlap. Experimentally, the coil overlap point is usually identified as the value of *c[η]* above which the specific viscosity begins to dramatically increase.

**Figure 1.**
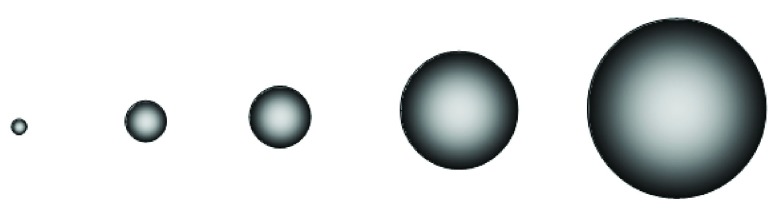
The hydrodynamic size of a hyaluronan chain depends on its molecular weight. Hyaluronan chains with molecular weight of (from left to right) 0.1, 0.5, 1, 3 and 6 million have hydrodynamic diameters of approximately 50, 140, 210, 400, and 600 nm, respectively in physiological saline solution. The diameter of a small globular protein would be on the order of a few nm. Adapted from Cowman and Matsuoka ^20^.

In order to estimate the concentration at which a HA solution might exceed coil overlap, we can consider the hydrodynamic size of the polymer at different molecular weights ( Figure 1) ^20^. Some example chain parameters are given below ( Table 1) ^20^. For HA with a molecular weight of 6 million, overlap requires a HA concentration of only about 320 µg/cm ^3^ (=2.5/7700). For HA with a molecular weight of 1 million, the coil overlap concentration would be about 1400 µg/cm ^3^. For comparison, the concentration of HA in human synovial fluid is usually 2000–3000 µg/ml, and the average molecular weight is close to 6 million, so the HA chains are well above the coil overlap point.

**Table 1.** Hydrodynamic size and intrinsic viscosity for hyaluronan of several different molecular weights, in physiological saline solution. The chain contour length, L, is calculated as M/M _L_, where M _L_ is the mass per unit length, approximately 401 nm ^-1^, for the sodium salt form. The intrinsic viscosity of hyaluronan is related to M by the equation [η] = 0.029 M ^0.80^. The specific volume of the polymer, V _s_, was obtained from the intrinsic viscosity, as [η]/2.5. Chain hydrodynamic diameter was approximated by the root mean square end-to-end distance, <r ^2^> ^1/2^, of the polymer chain, which is equal to ([η]M/Φ) ^1/3^, where Φ is the Flory constant with the empirical value of 2.1 x 10 ^23^. Coil overlap concentration was calculated as 2.5/[η], or equivalently, 1 / V _s_. Adapted from Cowman and Matsuoka ^20^.

M	*L* (nm)	*[η]* cm ^3^/g	*V _s_* cm ^3^/g	<r ^2^> ^1/2^ (nm)	c, for coil overlap (µg/cm ^3^)
					
1 × 10 ^5^	250	290	120	52	8600
5 × 10 ^5^	1250	1100	420	140	2400
1 × 10 ^6^	2500	1800	730	210	1400
3 × 10 ^6^	7500	4400	1800	400	570
6 × 10 ^6^	15000	7700	3100	600	320

**Figure 2.**
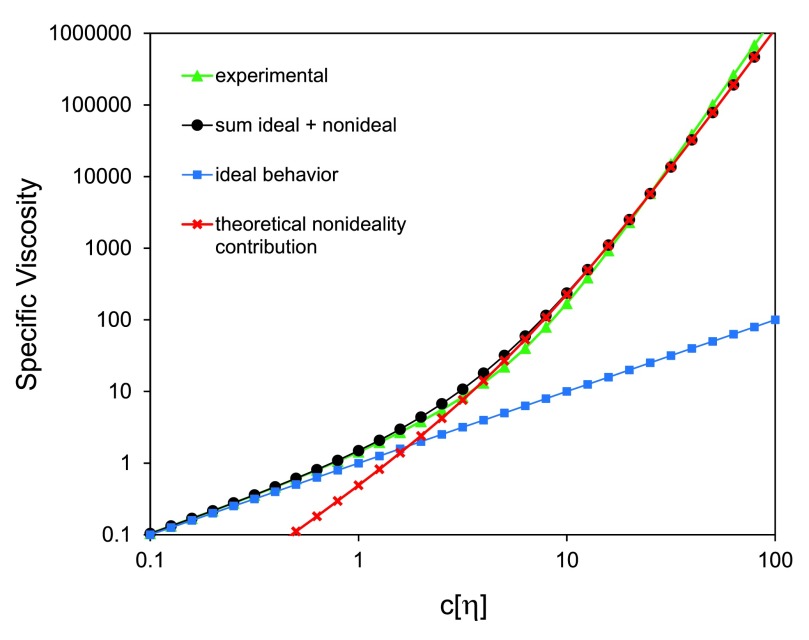
Specific viscosity of hyaluronan solutions, as a function of the concentration and intrinsic viscosity *[η]*. Experimental data for hyaluronan in physiological saline, plotted using the fitted equation *η _sp_* = *c[η]* + 0.42 *(c[η])* ^2^ + 7.77 x 10 ^-3^ *(c[η])* ^4.18^ reported by Berriaud and coworkers ^21^, shows a marked increase in viscosity with increasing concentration and intrinsic viscosity. (Note that this data represents low shear conditions, where hyaluronan chains are not distorted or aligned with flow.) The experimental data can be compared with predictions based on theory. For an ideal case in which the hyaluronan molecules act independently, the specific viscosity would simply be equal to the product *c[η]*. When the molecules become crowded, the effective concentration increases, leading to a significant nonideality contribution, predicted by the last three terms of the mutual macromolecular crowding equation, ( Equation 2 in text).

Based on the ideal model for HA in solution, specific viscosity should be equal to *c[η].* A comparison of the observed behavior of HA solutions to the ideal case ( Figure 2) shows that the ideal model is far from sufficient. Well below the coil overlap point of *c[η]=2.5*, HA solutions are already significantly non-ideal. Crowding between molecules increases the viscosity above that expected for the ideal solution.

### Real HA solutions show nonideality due to mutual macromolecular crowding, and greatly increased viscosity

When polymer molecules in solution begin to restrict the space available for movement of other chains, the solution is no longer dilute, and our simple model needs modification. In Figure 2, a comparison of the curve from experimental data ^21^ can be seen to deviate from the ideal case at values of *c[η]* well below the nominal coil overlap point of 2.5.

**Figure 3.**
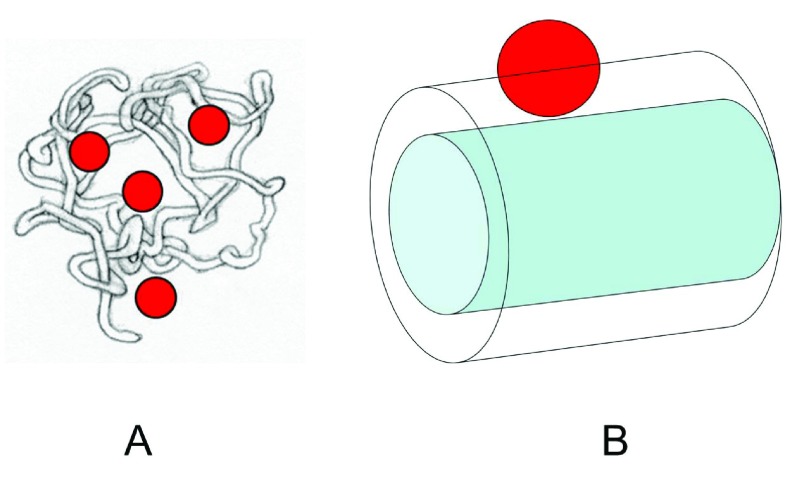
Model for steric exclusion of a globular protein by a hyaluronan molecule. **A** illustrates the ability of small globular proteins to penetrate most of the hydrodynamic domain of the hyaluronan polymer. **B** shows the size of the excluded volume for a globular protein in the presence of a segment of a linear polymer as a crowding agent. The cross section of the cylindrical excluded volume has a radius equal to the sum of the radius of the crowding polymer and the thickness of a cylindrical shell determined by the radius of the globular protein. This figure has been reproduced with permission from ^23^ Cowman *et al.* (2012) in Structure and Function of Biomatrix. Control of Cell Behavior and Gene Expression. Ed. E.A. Balazs, pp.45–66. Copyright 2012 Matrix Biology Institute.

We have developed a theory for crowding between flexibly coiled macromolecules like HA ^18, 22– 24^. It is based on the theory for gel filtration, developed by Ogston and Laurent ^25– 27^. The Ogston-Laurent theory for excluded volume provides a rational basis for understanding how proteins can be affected by a random suspension of fibers. A globular (spherical) protein is excluded from the space (a cylindrical shell) surrounding a fiber, by its own radius, and the thickness of the fiber. The center of the spherical protein defines its position, and the center cannot approach the fiber more closely than the sum of the radius of the sphere and the finite radius of the fiber. The probability of a protein finding space in a random suspension of such fibers (corresponding to the interior of a gel bead) is exponentially decreased as a function of the excluded volume. More fibers, or bigger proteins, mean less available space, and lower probability of being inside the beads. A similar picture can be imagined for the crowding of globular proteins by HA chains ( Figure 3).

**Figure 4.**
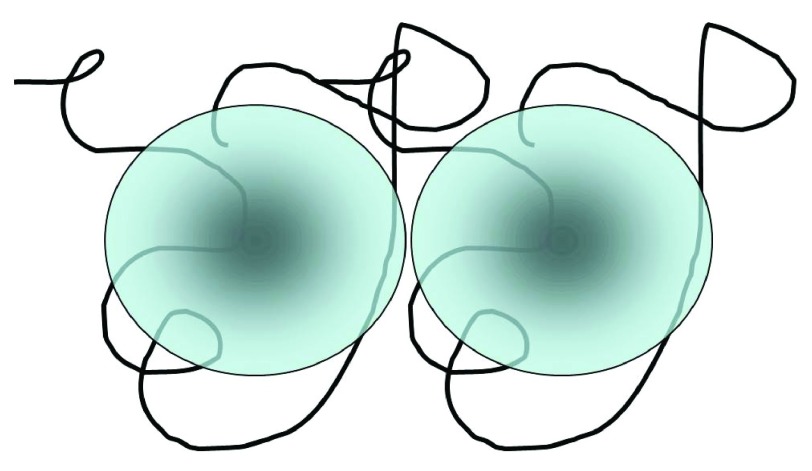
Model for mutual macromolecular crowding of hyaluronan molecules. The effective hydrodynamic domain of each chain is modeled as a sphere, the volume of which is dependent on the molecular weight to the 1.8 power. This figure has been reproduced with permission from ^23^ Cowman *et al.* (2012) in Structure and Function of Biomatrix. Control of Cell behavior and Gene Expression. Ed. E.A. Balazs, pp.45–66. Copyright 2012 Matrix Biology Institute.

We adapted the Ogston-Laurent excluded volume concept to the problem of mutual exclusion (mutual macromolecular crowding) that occurs between coiled HA chains in solution. Each chain crowds the others, by an amount related to the hydrodynamic volume it occupies, rather than just its physical chain length and thickness ( Figure 4).

The reduced probability of finding space for movement as a function of increased total concentration makes the effective concentration of the HA greater. The effective concentration is exponentially increased with HA real concentration and with the intrinsic viscosity. Since intrinsic viscosity is a measure of hydrodynamic volume, it is connected with molecular weight. The viscosity of HA solutions should then increase exponentially with concentration and molecular weight (as measured by intrinsic viscosity). We expanded the exponential term into a series. The first four terms of the series provide an excellent approximation of the observed specific viscosity. The first term is the ideal case, and the next three terms provide the nonideality contribution as shown in Figure 2. The sum of the two matches the experimental observation well ^18^.

$$\eta_{sp}=c[\eta ]\left(1+k^{\prime }c[\eta ]+\frac{{\left(k^{\prime }c[\eta ]\right)}^{2}}{2!}+\frac{{\left(k^{\prime }c[\eta ]\right)}^{3}}{3!}\right)\;k^{\prime }=0.4\;(2)$$

Now the extremely high viscosity of HA solutions can be successfully rationalized on the basis of mutual macromolecular crowding, which increases the effective concentration of the HA, and substantially increases the viscosity. There is no need to invoke intermolecular association or ordered structures of the HA molecules. It is also of great interest to note that, depending on the starting point on the curve, increasing or decreasing the HA concentration or intrinsic viscosity (and thus molecular weight) can have enormous impact (e.g., varying as the third or fourth power of the change). The absence of ordered structures in pure semi-dilute HA solutions is also supported by HA diffusion coefficients measured by the confocal Fluorescence Recovery After Photobleaching (FRAP) studies of Hardingham and coworkers ^28, 29^.

### Effect of temperature and pH on the viscosity of hyaluronan solutions

The large hydrodynamic volume of HA chains depends on the stiffness of the chain, which is due to steric hindrance to rotation about the linkages between sugar residues, and to the dynamically forming and breaking hydrogen bonds across those linkages. With increasing temperature, rotations about the linkages are easier, and the chains gain flexibility. This shrinks the molecular volume, and consequently reduces the viscosity. It is possible to predict the extent of viscosity reduction, based on Equation 2, and the known dependence of the intrinsic viscosity on temperature. Data from Cleland ^30^ and Fouissac, Milas, and Rinaudo ^31^ show that the intrinsic viscosity of high molecular weight HA is decreased by about 25% as the temperature is increased from 25° to 65°C. Hoefling *et al.* ^32^ showed that incorporating the 25% decrease in intrinsic viscosity into Equation 2 above gave an excellent prediction of the 2–3 fold experimental change in specific viscosity of semidilute HA solutions over that temperature range. There is no need to propose a change from an ordered conformation to a disordered one, because a modest increase in chain flexibility explains the marked solution viscosity change with temperature.

The viscosity of HA solutions is not very sensitive to pH in the physiological range. At very high pH, above about pH 11, the rotational freedom at the glycosidic linkages is greatly increased due to breakage of residual hydrogen bonds, and the chain volume shrinks, reducing the solution viscosity ^33– 35^. At low pH of about 2.5, at physiological ionic strength, an interesting viscoelastic putty (nearly like a gel) is formed as a result of interchain association ^33, 35– 37^. But between pH values of 6.5–8.0, the expansion of the hyaluronan chains is nearly constant, and the intrinsic viscosity is not changed ^38^.

### The shear rate dependence of the viscosity, and the viscoelasticity of HA solutions, depend on the relaxation time of the molecule, while relaxation time depends on HA concentration and molecular weight

**Figure 5.**
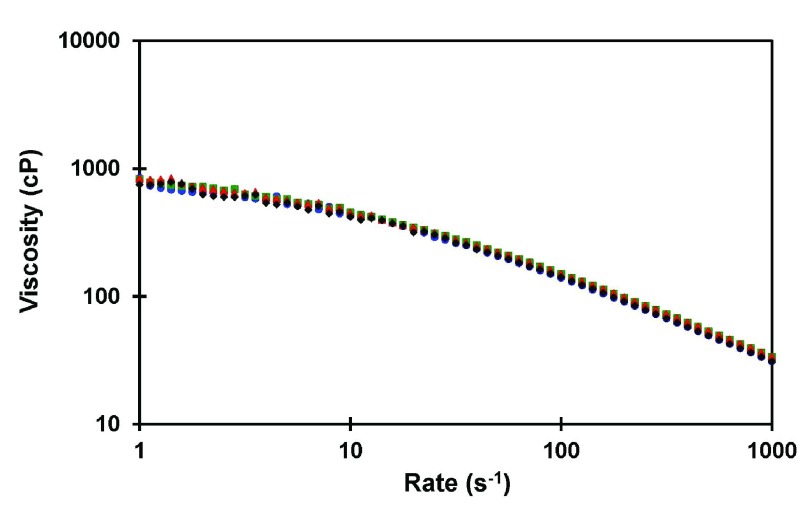
Shear rate dependence of the viscosity of a polydisperse hyaluronan sample (viscosity-average molecular weight = 1.7 million) at a concentration of 5 mg/ml in PBS at 25°C. Data from three consecutive runs are shown. This figure has been reproduced with permission from ^39^ Cowman *et al.* (2011) Anal. Biochem., 417, 50–56. Copyright 2011 Elsevier Inc.

When a solution containing flexible polymers is flowing, the molecules can become distorted and stretched in the direction of the flow. The more slowly the molecules recover to their undisturbed shapes, relative to the rate of shear, the more they become aligned with the flow. The aligned molecules have a reduced contribution to the solution viscosity. In steady shear conditions, the solution viscosity is highest when the rate of shear is low, and molecules can reorient and relax to the undisturbed shape as rapidly as they move. But with increasing shear rate, the molecules cannot relax fast enough, and the viscosity drops. Figure 5 shows a typical example of the viscosity of a semi-dilute solution of HA in physiological solution ^39^.

This shear rate effect is seen for both dilute and semi-dilute solutions. In dilute solutions, the relaxation time depends on the molecular volume (proportional to the molar volume, *[η]M)* and the solvent viscosity. The more viscous the solvent, the longer the time needed for relaxation. In semi-dilute, or crowded solutions, the relaxation time is much more strongly increased because molecules must find space to move past each other. Again, the probability of finding space is exponentially related to the excluded volume. The higher the molecular weight, or the higher the concentration, the longer the relaxation time, and the more dramatic the loss of viscosity (shear thinning) with increasing shear rate ^18^.

**Figure 6.**
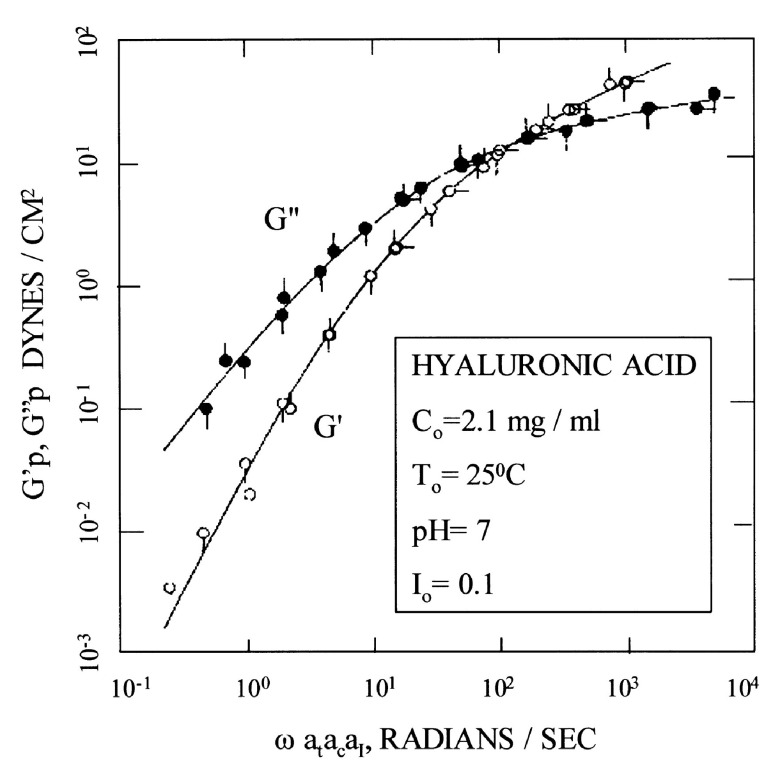
Master curves for the elastic modulus (G') and the viscous modulus (G") of a solution of HA with a molecular weight of 2.8 x 10 ^6^ as a function of the frequency of displacement. This figure has been reproduced with permission from ^40^ Gibbs *et al.* Biopolymers **1968**, 6, 777–791. Copyright 1968 John Wiley & Sons, Inc.

Another consequence of the long relaxation time of large HA molecules in semi-dilute solutions is a transition from viscous behavior to elastic behavior as a function of increasing rate of deformation ( Figure 6) ^40^. If a solution is cyclically deformed, then slow rates allow the molecules to keep up with changes and flow. But rapid cyclic deformation does not allow the molecules to relax in shape, and instead they behave elastically, stretching and recoiling without flow. This behavior is called viscoelastic. For HA, it plays an important role in its protection of the articular joints under rapid motion. For HA in other tissues such as fascia, it can inhibit flow if the concentration and molecular weight are large enough that elastic behavior dominates under normal rates of motion.

### Effect of viscosity on lubricating function of HA solutions

**Figure 7.**
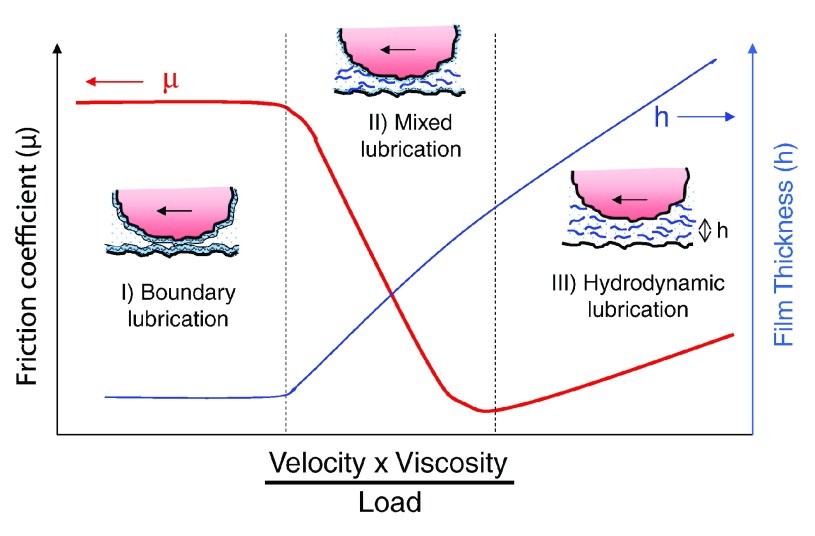
Friction coefficient plotted as a function of fluid viscosity and shear velocity divided by load (Stribeck curve) with corresponding lubrication film thickness. The schematic shows boundary, mixed, and hydrodynamic lubrication regimes. This figure has been reproduced with permission from ^41^ Coles *et al.* (2010) Curr. Opin. Colloid Interface Sci. 15, 406–416. Copyright 2010 Elsevier Ltd.

The three main modes of lubrication are boundary, fluid film or hydrodynamic, and mixed. In boundary mode lubrication, surface-to-surface contact occurs between articulating surfaces, and molecules bound to the surface mediate friction. In fluid film lubrication, a thick (relative to the surface roughness of the articulating surfaces) viscous fluid film supports the load and separates the surfaces allowing motion with little resistance to shear. Mixed mode lubrication is where both boundary and fluid film mode lubrication are operative. The conditions under which each mode operate are classically defined by a Stribeck curve ( Figure 7), which demonstrates how a friction coefficient (µ = friction force divided by normal force) varies with (velocity × viscosity/load) ^41^. Boundary mode lubrication occurs at the left end of the curve (low velocity and high loads with a small film thickness), whereas fluid film lubrication occurs at right end of the curve (high velocity and low loads with a large film thickness). While this curve was generated using classic hard, non-porous, engineering materials (e.g. steel), and may not be completely applicable to soft, porous, hydrated tissues or materials ^42^, it is still useful in understanding general conditions under which different modes of lubrication are operative.

**Figure 8.**
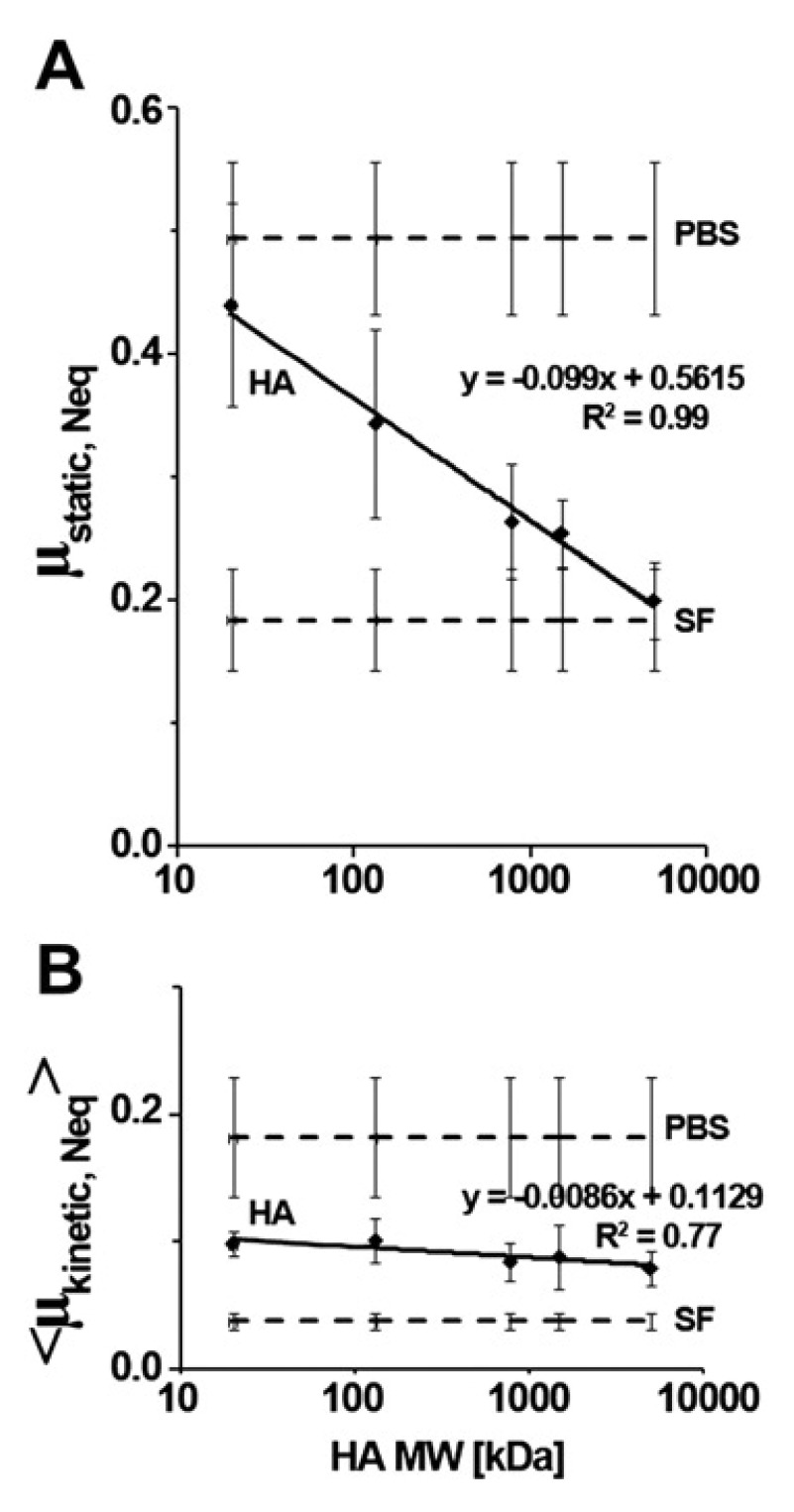
Dependence of the cartilage boundary lubricating properties of hyaluronan alone at 3.33 mg/ml on molecular weight. Regression lines are shown for ( **A**) mean static µ _static, Neq_ friction values at pre-sliding duration, T _ps_ = 1,200 s, and ( **B**) mean kinetic <µ _kinetic, Neq_> friction values at T _ps_ = 1.2 s obtained vs log molecular weight of hyaluronan. Mean values in phosphate-buffered saline (PBS) and synovial fluid (SF) are shown for reference. This figure has been reproduced with permission from ^43^ Kwiecinski *et al.* (2011) Osteoarthritis Cartilage 19, 1356–1362. Copyright 2011 Osteoarthritis Research Society International.

The viscosity of HA solutions can affect the mode of lubrication in which HA reduces friction as its relative effectiveness in reducing friction at tissue biointerfaces. For example, in a boundary mode of lubrication at a cartilage-cartilage biointerface, onto which HA is able to bind, relative effectiveness of friction reduction (especially static friction, the resistant to start up motion) has been shown to be dependent on the molecular weight of HA, with higher molecular weight resulting in lower friction ( Figure 8) ^43^. This has been speculated to be due to a ‘viscous boundary layer’ of HA at the surface of cartilage ^44^.

Conversely, in fluid film lubrication where a thick film of HA separates articulating surfaces, friction would be predicted to increase with viscosity, potentially reaching very high levels. In deep fascia, the thickness of the HA-containing fluid layer is apparently on the order of tens of microns, which is large compared with the diameter of the molecules and even the roughness of the surfaces. In such a case, if HA concentration and/or molecular weight are high, the resistance to flow due to high viscosity can negatively affect lubrication.

### Lubricin can decrease the viscosity of crowded high molecular weight hyaluronan solutions

**Figure 9.**
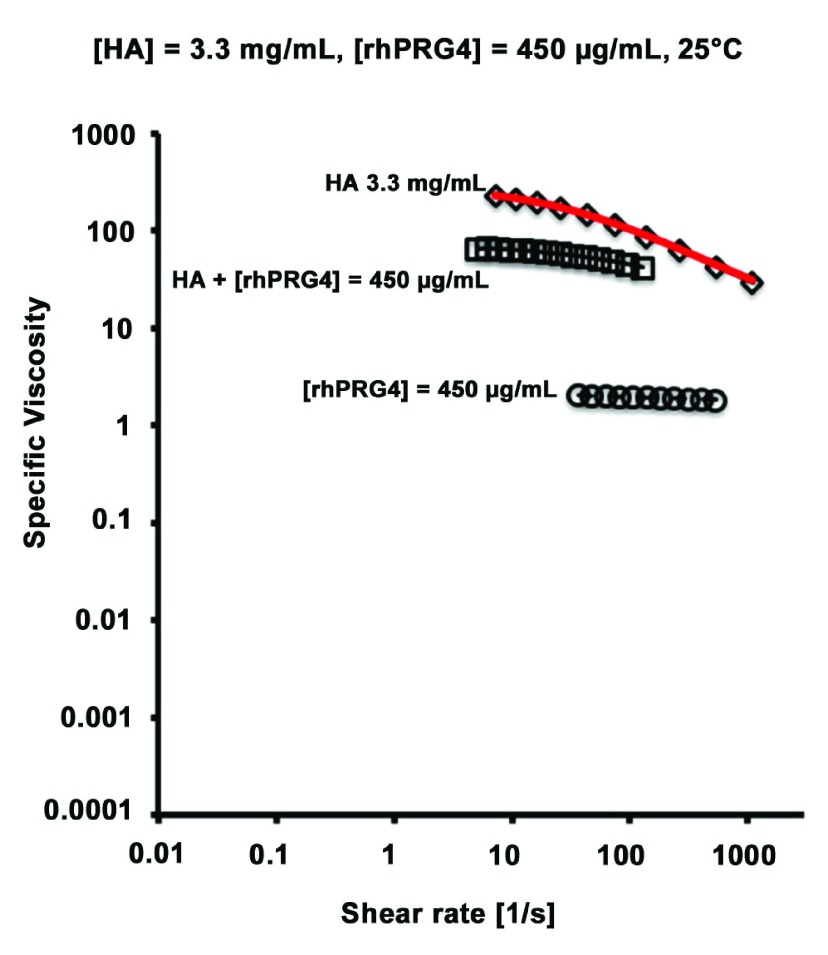
Lubricin (rhPRG4) can reduce the viscosity of HA (weight-average molecular weight of 1.5 million) solution when both are present at concentrations found in normal human synovial fluid. Shear rate dependent specific viscosity at 25°C of hyaluronan at 3.3 mg/ml alone and with 450 μg/ml rhPRG4, shown in black. Predicted specific viscosity (experimental hyaluronan + experimental rhPRG4) shown in red. This figure has been reproduced with permission from ^45^ Ludwig *et al.* (2014) Biorheology 51, 409–422. Copyright 2014 IOS Press and the authors.

Lubricin is a lubricating mucin-like glycoprotein present in various body fluids, such as synovial fluid (essentially a 2–3 mg/ml solution of high molecular weight HA), that can alter the viscosity of HA solutions. We have shown that lubricin is able to reduce the viscosity of a high molecular weight HA solution when both components are present at physiological concentrations ( Figure 9) ^45^, potentially by binding and shrinking the hydrodynamic domains of HA molecules, enabling them to flow more easily. In cases where HA concentration becomes high and viscous flow is reduced, lubricin could facilitate increased motion and thus decreased friction.

### HA is increased in concentration during inflammation, and can be covalently modified

A common observation in inflamed tissues is an increase in the concentration of HA ^2^. The HA content of injured skeletal muscle is known to be elevated ^46^. Stecco *et al.* ^17^ documented, with a highly specific HA-binding peptide, the deposition of HA inside the loose connective tissue in three different fasciae of the body: fascia lata, rectus abdominis sheet and sternocleidomastoid (SCM) fascia. Stecco *et al.* ^20^ also documented an increase of the thickness of the loose connective tissue in the SCM fascia in patients complaining of chronic neck pain syndrome. If the HA content of fascia is increased, the viscosity and elasticity of the HA-containing fluid would be increased, and its fluid film lubricating properties reduced.

Possibly more important might be the covalent modification of HA by heavy chain domains derived from plasma inter-α-inhibitor (IαI). An increase in the expression of TSG-6 protein is commonly observed during inflammation. TSG-6 acts catalytically to transfer heavy chain (HC) domains from the chondroitin sulfate chain of IαI to HA ^47– 49^. This transfer is normally a protective function that can stabilize the pericellular coat of cells. The HC domains can dimerize, and effectively act to hold HA chains noncovalently together ^50, 51^. It could be imagined that the HA, modified by HC, becomes gel-like and immobile in the deep fascia. HC-modified HA can also be found aggregated into fibers or cables ^52, 53^. An increase in both HA and TSG-6 has been reported in cultured vascular smooth muscle cells subjected to mechanical strain ^54^, and proliferating smooth muscle cells in rat neointima after injury express high levels of TSG-6 ^55^. Recently, an increase in HA, TSG-6, and HC-modified HA was observed in damaged mouse skeletal muscle tissue ^46^.

## Discussion

The fascia assumes a fundamental role with its two components: dense connective tissue (collagen fibers type I and III) and loose connective tissue (adipose cells, GAGs (glycosaminoglycans), and HA). HA is an important component of the loose connective tissue in fascia. In this review, we have considered the physico-chemical properties of HA solutions, and how they depend on factors such as concentration, molecular weight, and modification by covalent linkage to HC derived from IαI, or noncovalent interactions with proteins such as lubricin.

### Effect of immobility on concentration of HA and muscle structure

Immobilization of a limb or body segment can lead to an increase in the concentration of HA within and between the fascial and muscular compartments, which can increase the fluid viscosity. The increased fluid viscosity within the loose connective tissue can in turn decrease the gliding between the layers of collagen fibers, which may be perceived by the subject as stiffness ^56^. Changes documented in rat soleus muscle due to one week of immobilization include increase in HA concentration and shortening of sarcomere length ^57^. These changes were postulated to increase the number of cross bridges attached during contraction ^58, 59^. In the early stages of this process, the arrangement of collagen fibrils in the endomysium may remain longitudinal, however, by about 4 weeks, the collagen fibrils became arranged circumferentially, which signals pre-contracture. Thus subtle changes in the turnover of HA and in the properties of the extracellular matrix with immobility can lead to structural and eventually functional changes in the muscles with significant consequences on movement ^60^.

The interdependence of mechanoreceptor activation and viscoelasticity of the surrounding tissue has been previously noted ^61– 65^. Due to the fundamental role of HA in determining the viscoelasticity of fluids in soft connective tissues, its alteration could therefore modify the activation of the receptors, producing non-specific musculoskeletal pain.

The increased HA content of fascia and the underlying muscle may result from increased HA synthesis, due to a stimulation of the fibroblast-like cells that were previously suggested to be the biosynthetic source of hyaluronan ^17^. It may also reflect impaired turnover via flow toward the lymph. A high HA concentration would increase the viscosity of the HA-containing fluids. When the viscosity of the fluid in the loose connective tissue increases due to increased HA concentration or its covalent modification, the dense connective tissue can spread the stiffness throughout the surrounding areas, driving even further the sensation of muscle stiffness. Deep friction manipulation may aid outflow of HA if the effective shear rate within the fluid layers generates a drop in the viscosity. This may explain the reduced perception of stiffness that is reported by both therapist and patient during this manual treatment. This review suggests a basis for the typical finding in manual therapy: the more chronic is the stiffness, the higher the concentration of HA may be, and the greater the effort and time required for manual treatments ^65^.

### Effect of hyaluronidase on HA

There are a number of HA-cleaving enzymes ^6^. For medical applications, a preparation containing a recombinant fragment of human PH20 hyaluronidase is currently available. It hydrolyzes HA (and susceptible linkages in chondroitin sulfate glycosaminoglycans) by splitting the glycosidic bond between C1 of an N-acetylhexosamine moiety and C4 of a glucuronic acid moiety. It reduces the molecular weight, and would be expected to lower the viscosity of the extracellular matrix fluid and thus make outflow easier. It can also disrupt aggregates or gel made by HA crosslinked via HC chains. The products of enzymatic cleavage may include small oligosaccharides of HA, which have been reported to trigger specific inflammatory responses and have a pro-inflammatory effect ^66, 67^. That possibility has been disputed ^68^. In any case, the presence of fragments generated by the action of hyaluronidase is expected to be short lived as restored flow can wash the small polymers away. Hyaluronidase is used primarily as a dispersion agent, but may now be considered for use in conditions where altered viscosity of the fascia is desired, such as in muscle stiffness.

## Conclusion

The physico–chemical properties of HA are modulated by its concentration, molecular weight, solvent ionic composition, temperature, and covalent or noncovalent binding of proteins and other species. If HA is forced to exist in a highly crowded environment, or more generally if its density within the loose connective tissue inside the fascia is increased as a result of injury or other pathological process, the behavior of the whole deep fascia and of the underlying connective tissue epimysium and perimysium could be compromised. Treatments that address the role of HA may hold promise.
