# Supplementary material for: Viscoelastic Properties of Hyaluronan in Physiological Conditions
Source: F1000Res. 2015 Aug 25;4:622. [Version 1] doi: 10.12688/f1000research.6885.1 (PMC4648226; doi:10.12688/f1000research.6885.1)
Supplement: Peer review history

# Peer review: Referee response for version 1

**DOI:** 10.5256/f1000research.7412.r10133

**Published:** 2015-09-14

**Authors:** John Sandy, Anna Plaas

**Affiliations:** 1 Department of Biochemistry, Rush University Medical Center , Chicago, IL, USA; 2 Biochemistry, Internal Medicine, Rush University Medical Center, Chicago, IL, USA

This expertly constructed review article on hyaluronan (HA) provides an in-depth analysis of the relationship between its structural and physicochemical properties. The article also contains a well-informed update on the effects of modifications, such as protein binding, cross-linking and molecular weight on these properties. In particular, this is discussed in relation to its role in regulating the viscoelasticity of loose connective tissues (fascia) required for effective organ separation. For example, the authors point out that abnormally high HA-dependent viscosity in muscle fascia can lead to local and more generalized stiffness, and potentially mechanosensor-mediated pain. Indeed, this section could be expanded to underline the importance of future research on the effects of structural modifications on catabolic processing of HA, both within the extracellular matrix and in fluid compartments. On the general topic of hyaluronan-mediated effects at interfaces, readers will also find interesting the papers from Richter and colleagues who are examining the effects of end-grafted HA on the properties of glycoconjugate cell coats ( Richter, *et al*., 2007), lipid membranes ( Attili, *et al*., 2012), nanoparticles ( Bano, *et al*., 2015) and pulmonary surfactants ( Lopez-Rodriguez, *et al*., 2013).

We have read this submission. We believe that we have an appropriate level of expertise to confirm that it is of an acceptable scientific standard. Competing Interests: No competing interests were disclosed.

We have read this submission. We believe that we have an appropriate level of expertise to confirm that it is of an acceptable scientific standard.

# Peer review: Referee response for version 1

**DOI:** 10.5256/f1000research.7412.r10135

**Published:** 2015-09-02

**Authors:** Timothy Hardingham

**Affiliations:** 1 Wellcome Trust Centre for Cell-Matrix Research, Faculty of Life Sciences, University of Manchester, Manchester, UK

This is an excellent review of the current biophysics and biology of hyaluronan. It presents an analysis of the complex non-ideal behaviour of hyaluronan characterised by different biophysical techniques and interprets this technical information in a way that enables the non-specialist to understand the consequences these properties have in biology. It's up to date and presents a very readable insight into the complex behaviour of this essentially simple biopolymer.

I have read this submission. I believe that I have an appropriate level of expertise to confirm that it is of an acceptable scientific standard.
